# Closing the loop between brain and electrical stimulation: towards precision neuromodulation treatments

**DOI:** 10.1038/s41398-023-02565-5

**Published:** 2023-08-14

**Authors:** Ghazaleh Soleimani, Michael A. Nitsche, Til Ole Bergmann, Farzad Towhidkhah, Ines R. Violante, Romy Lorenz, Rayus Kuplicki, Aki Tsuchiyagaito, Beni Mulyana, Ahmad Mayeli, Peyman Ghobadi-Azbari, Mohsen Mosayebi-Samani, Anna Zilverstand, Martin P. Paulus, Marom Bikson, Hamed Ekhtiari

**Affiliations:** 1 Department of Psychiatry & Behavioral Sciences, University of Minnesota, Minneapolis, MN USA; 2 Department of Biomedical Engineering, Amirkabir University of Technology, Tehran, Iran; 3 Department of Psychology and Neuroscience, Leibniz Research Center for Working Environment and Human Factors, Dortmund, Germany; 4 Bielefeld University, University Hospital OWL, Protestant Hospital of Bethel Foundation, University Clinic of Psychiatry and Psychotherapy, and University Clinic of Child and Adolescent Psychiatry and Psychotherapy, Bielefeld, Germany; 5 Neuroimaging Center, Focus Program Translational Neuroscience, Johannes Gutenberg University Medical Center Mainz, Mainz, Germany; 6 Leibniz Institute for Resilience Research, Mainz, Germany; 7 School of Psychology, Faculty of Health and Medical Sciences, University of Surrey, Guilford, UK; 8 Department of Psychology, Stanford University, Stanford, CA USA; 9 MRC CBU, University of Cambridge, Cambridge, UK; 10 Department of Neurophysics, MPI, Leipzig, Germany; 11 Laureate Institute for Brain Research, Tulsa, OK USA; 12 School of Electrical and Computer Engineering, University of Oklahoma, Tulsa, OK USA; 13 University of Pittsburgh Medical Center, Pittsburg, PA USA; 14 Department of Biomedical Engineering, Shahed University, Tehran, Iran; 15 Iranian National Center for Addiction Studies, Tehran University of Medical Sciences, Tehran, Iran; 16 City University of New York, New York, NY USA

**Keywords:** Neuroscience, Psychology

## Abstract

One of the most critical challenges in using noninvasive brain stimulation (NIBS) techniques for the treatment of psychiatric and neurologic disorders is inter- and intra-individual variability in response to NIBS. Response variations in previous findings suggest that the one-size-fits-all approach does not seem the most appropriate option for enhancing stimulation outcomes. While there is a growing body of evidence for the feasibility and effectiveness of individualized NIBS approaches, the optimal way to achieve this is yet to be determined. Transcranial electrical stimulation (tES) is one of the NIBS techniques showing promising results in modulating treatment outcomes in several psychiatric and neurologic disorders, but it faces the same challenge for individual optimization. With new computational and methodological advances, tES can be integrated with real-time functional magnetic resonance imaging (rtfMRI) to establish closed-loop tES-fMRI for individually optimized neuromodulation. Closed-loop tES-fMRI systems aim to optimize stimulation parameters based on minimizing differences between the model of the current brain state and the desired value to maximize the expected clinical outcome. The methodological space to optimize closed-loop tES fMRI for clinical applications includes (1) stimulation vs. data acquisition timing, (2) fMRI context (task-based or resting-state), (3) inherent brain oscillations, (4) dose-response function, (5) brain target trait and state and (6) optimization algorithm. Closed-loop tES-fMRI technology has several advantages over non-individualized or open-loop systems to reshape the future of neuromodulation with objective optimization in a clinically relevant context such as drug cue reactivity for substance use disorder considering both inter and intra-individual variations. Using multi-level brain and behavior measures as input and desired outcomes to individualize stimulation parameters provides a framework for designing personalized tES protocols in precision psychiatry.

## Introduction

Brain-based “targets” for interventions are commonly used to describe neuronal processes, circuits, or molecular structures in human or animal nervous systems. The activities of these brain-based targets can be modulated by a specific intervention to produce therapeutic effects. In-vivo and in-vitro brain mapping tools in human or animal studies uncover complex relevant neural mechanisms to modulate specific targets during an intervention [1–6]. In-silico studies, including machine learning, computational modeling, and simulation approaches, have also provided new mechanistic insights to assess the efficacy of interventions to modulate a brain-based target [7–9]. In thinking about brain-based neural interventions and trying to use those to change a brain state and ultimately alter a clinical/behavioral outcome, it would be necessary to define a target that is specific and amenable to modulation, and in which its modulation will impact behavior (e.g., by changing functional activity/connectivity in the brain target that may affect clinical/behavioral outcomes as the NIMH Research Domain Criteria (RDoC) suggests [10]).

Brain stimulation methods are considered as interventions that can engage such a target based on the stimulation protocol; open-loop or closed-loop paradigm [11]. In open-loop interventions, there is no feedback or control signal to change or adjust stimulation parameters based on the ongoing response (i.e., the output of the stimulation has no effect on the stimulation protocol). Because little is known about the stimulation effects in the long-term, in open-loop paradigms, stimulation dose (including intensity, frequency, and phase difference) is selected based solely on previous empirical evidence and remains fixed during the experiment without considering the non-stationary nature of brain activities. In contrast, adaptive closed-loop intervention employs brain mapping tools to record the mechanism of action in a predefined target, and stimulation parameters are iteratively defined based on the ongoing neurophysiological variations in the targeted brain areas that may help to solve limitations of the open-loop interventions and increase the efficacy of the stimulation treatments [11, 12].

**Fig. 1:**
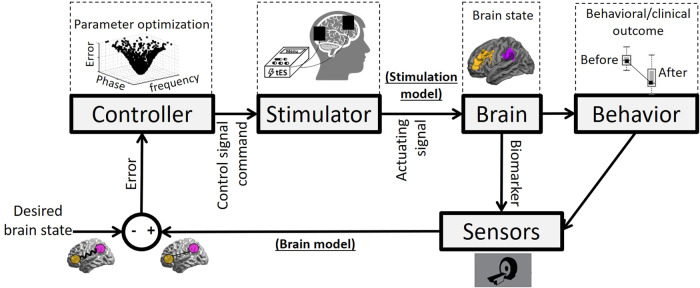
Block diagram of closed-loop stimulation: an engineering perspective. Different parts of this system are constituting the output: brain state or behavior, Desired brain state: a predefined reference. Measured brain state (measurement): measured/quantified brain state known as a biomarker (as an indirect indicator of brain state; e.g., frontoparietal synchronization). Comparator: an algorithm that compares a measured brain state with a predefined desired brain state and sends the comparison result (named Error signal) to the controller. When there is no difference between desired and measured brain states, the comparator output is zero. Therefore, the controller input is also zero, which means that there is no need to change the stimulation parameters. Controller: an optimization algorithm that receives the difference between desired and measured brain states and tries to find optimum values of stimulation parameters based on minimizing differences between the desired and measured brain states. Stimulator: a neurostimulation device such as transcranial electrical stimulator (tES) that adjusts its parameters (e.g., phase and frequency) based on information received from the controller. Brain: the plant under-stimulation. Sensor: a hardware or device that records/quantifies the current brain state (e.g., fMRI system). Control signal/command: signal/command to titrate stimulation dose automatically. Actuating signal: electrical current stimulation signals applied to the brain. Behavior: the loop can be extended to behavior, and instead of target engagement biomarkers, a treatment response biomarker is recorded (e.g., drug craving self-report). Brain model (generated by sensors): A model that links the behavioral and clinical outcome and biomarker to a disease mechanism and defines the dynamic targets for engagement and change. Stimulation model: A model that includes technical properties of the stimulator and its mechanism of action to predict the optimized protocol based on the inputs from the stimulation model.

Control-oriented models for closed-loop applications in the area of neuroscience provide new opportunities for brain stimulation devices that have the ability to affect the brain in order to drive the brain process from a current state to a desired state [13]. A basic block diagram of a closed-loop system for dose titration is shown in Fig. 1. In this simple engineering perspective, as a brain-based closed-loop neuromodulation system, the brain is the plant that has a particular state. Brain state can be detected via signals recorded from the brain which are known as biomarkers (i.e., an electrophysiological or biological correlate of the neurological condition to be targeted). When a biomarker acts as a proxy measure of an intervention target, then it can be named as a “treatment response” biomarker [14] as we intend by our definition of biomarker here and afterward. In this closed-loop structure, biomarkers that are recorded via specific sensors and measures (like blood oxygenation level-dependent (BOLD) signals in functional magnetic resonance imaging (fMRI) or voltage changes in certain frequencies in electroencephalography (EEG)) are constantly monitored as a proxy of something that might be corresponding to the current brain state based on the “brain model”. This actual brain state is compared with the desired brain state and the difference (expressing how similar the current brain state is to be predefined desired brain state) will be sent to a controller to optimize the stimulation parameters. Through an optimization algorithm, stimulation parameters (e.g., stimulation intensity or frequency) are adjusted and tailored by the fluctuations of the error signal with the aim of regulating the current brain state. Then, the brain will be stimulated based on the “stimulation model” that includes the technical properties of the stimulator and its mechanism of action (in addition to transcranial electrical/magnetic stimulation (tES/TMS) technologies, this closed-loop system can be applied to other types of stimulators like deep brain stimulation (DBS) or transcranial focused ultrasound). Brain state alterations will be measured again indirectly via the biomarker, and then this loop is repeated until a predefined error threshold is reached. In addition to optimizing the similarity between the ongoing spatial pattern of activity and desired brain state, the optimization can also search for the optimum target using both brain and stimulation models. Furthermore, this neural-based paradigm can be extended to behavioral/clinical outcomes as well. Preliminary results with invasive (e.g., DBS) [15, 16] and noninvasive (e.g., transcranial focused ultrasound [17], tES, or TMS) [18–20] methods suggest the potential benefits of this closed-loop technology.

Recent advances in combining noninvasive brain stimulation with brain mapping tools like fMRI provided more effective stimulation protocols by informing stimulation parameters with functional brain activity/connectivity [21]. For example, the Stanford neuromodulation therapy (SNT) paradigm provided by Cole et al. [22], reported that individualized functional connectivity-guided targeting using resting-state fMRI in a high-dose intermittent theta burst stimulation (iTBS) trial was more effective than sham stimulation for treatment-resistant depression [22]. However, it is not clear that this improvement is due to the individualized connectivity-based targeting or several of the other novel aspects of the protocol (e.g., a large number of sessions every day or adjusted stimulation intensity) [22]. In addition to offline optimization of the stimulation protocols using fMRI data, real-time fMRI (rtfMRI) are enabling us to address closed-loop optimization based on the variability of the instantaneous brain state at the time of stimulation [23]. As defined by Sulzer et al, “rtfMRI is any process that uses functional information from an MRI scanner where the analysis and display of the fMRI keep pace with data acquisition” [24]. The organization of this document centers around integrating tES (as an intervention to modulate a target) with rtfMRI (to record target engagement or treatment response biomarkers). (1) We start by describing how fMRI could contribute as a potential measure of brain state. (2) This is followed by discussing the accumulated knowledge on the opportunities and challenges involved with using rtfMRI in other closed-loop systems (fMRI-neurofeedback). (3) We further discuss integrating fMRI data with tES. (4) We then explain how closing the loop between the brain and the stimulator can help to optimize tES (closed-loop tES-fMRI). (5) We then introduce potential benefits and challenges in the experimental design of closed-loop tES-fMRI studies. (6) There is no published clinical trial with closed-loop tES-fMRI so far (end of Jun 2023), so, lastly, through a conceptual example, we explain how closed-loop tES-fMRI can be designed as an intervention in a clinical trial.

### fMRI as a measure for brain state

fMRI is suited for studying brain states during a brain-based intervention program. Compared to other noninvasive brain imaging tools, such as electro- (EEG) or magnetoencephalography (MEG), fMRI provides a higher spatial resolution and whole-brain coverage to detect the specific anatomical regions affected by the intervention. Despite its limitations such as lower temporal resolution compared to EEG/MEG signals, and although there are major obstacles to using fMRI for drug development and clinical trials, fMRI has been used in a number of previous studies to identify neural targets and enhanced the development of novel therapeutic interventions and drugs in pre-clinical human studies [25–30]. For example, fMRI measures are being used successfully as treatment response biomarkers in drug development clinical trials [25]. As most new drug candidates which were successful in animal studies ultimately fail to engage the proposed target in humans and show meaningful responses in clinical applications, a fast-fail trial approach was proposed, in which fMRI is employed as a treatment response biomarker before moving for larger clinical trials. For instance, in the first implementation of a fast-fail trial via task-based fMRI, a hypothesis that k-opioid receptor (KOR) antagonism would enhance ventral striatal activation (target engagement important for reward processing and anhedonia treatment) was tested, and the study results established the proof-of mechanism for the KOR antagonism as a potential treatment for anhedonia [26].

Besides the drug development process, fMRI data are also used for assessing target engagement in cognitive interventions (treatment response biomarkers). The fMRI signal can provide indirect evidence for brain states if a biologically plausible link can be established between the fMRI response and cognitive interventions. As an example, among many others, a longitudinal fMRI study examined brain activity alterations following cognitive-behavioral treatment in a group of patients with posttraumatic stress disorder (PTSD) compared to healthy controls [27]. Reduced PTSD symptom severity from pre- to post-treatment was significantly correlated with reduced functional activity in the subgenual anterior cingulate cortex and hippocampus during task-based fMRI [27]. In accordance, a meta-analysis of fMRI studies on cognitive reappraisal interventions in mood and anxiety disorder patients revealed the effectiveness of the intervention for modulating the specific pattern of dysfunctional brain activation during cognitive reappraisal [28]. These findings suggest that neural activation or functional connectivity patterns identified in fMRI data may act efficiently as brain-derived measures or biomarkers of target engagement and treatment response in different types of interventions [28–31].

### fMRI in real-time closed-loop systems

Beyond what we discussed above on the applications of a static or averaged fMRI signal as a biomarker, the continued advances in MR imaging systems and experimental sophistication with the BOLD signal have led to the use of rtfMRI to decode the dynamic brain state and use it as a real-time brain-based biomarker for therapeutic applications [32, 33]. fMRI-guided neurofeedback as a potential treatment option for different psychiatric and neurologic disorders has been increasingly explored to help individuals to learn how to upregulate or downregulate the hemodynamic activity of a targeted brain region/network. In rtfMRI-neurofeedback, participants are trained to self-modulate a target and engage the target voluntarily based on visualization of the level of target engagement (fMRI feedback). Hence, with respect to the block diagram in Fig. 1, the controller and optimization algorithm work based on self-regulation. Simultaneously, fMRI data allow subjects to learn how to volitionally modulate brain activation with the goal to regulate it (i.e., subjects are both stimulator and controller in a self-regulated closed-loop system). Subjects are instructed about the strategies that could be used to control the fMRI feedback signal they will see. However, it still remains an open question whether this training for target engagement is feasible for all patients and eventually translates into meaningful long-term behavioral/clinical outcomes [34].

Despite promising results for the application of rtfMRI-neurofeedback in psychiatric disorders, there are no FDA-approved rtfMRI-neurofeedback trials for a specific neuropsychiatric disease, and there are methodological challenges in its implementation. Nonetheless, in a recent meta-analysis that investigated the influence of rtfMRI-neurofeedback on brain and behavioral outcomes in 17 studies and 105 effect sizes for psychiatric disorders, it was found that rtfMRI-NF produced a medium effect size on neural activity during training (Hedges’ *g* = 0.59, 95%), and small effect sizes for behavioral outcomes (symptoms Hedges’ *g* = 0.37, cognition Hedges’ *g* = 0.23) [35]. Another meta-analysis, including thirty-one clinical trials focusing on psychiatric disorders, that evaluated the efficacy of rtfMRI-neurofeedback on psychiatric symptoms revealed a large effect size for neurofeedback training on depressive symptoms right after the training (Hedges’ *g* = 0.81) and at follow-up (Hedges’ *g* = 1.19), as well as a moderate effect on anxiety (Hedges’ *g* = 0.44) and emotion regulation (Hedges’ *g* = 0.48) [36].

However, rtfMRI-neurofeedback efficacy varies between studies and participants and many factors can influence its success rate. A big data machine learning mega-analysis approach, using 608 participants from 28 independent experiments, with a classification accuracy of 60% showed that two factors significantly influenced rtfMRI-neurofeedback performance: a pre-training no-feedback run before neurofeedback training, and neurofeedback training of patients as compared to healthy participants [37]. These results, combined with proof-of-concept studies such as connectivity-based rtfMRI-neurofeedback for reducing negative thinking in depression [38] and tobacco use disorders [39], underscore the potential benefits of rtfMRI-neurofeedback, however, more research is needed to determine how it works, for whom, and under what circumstances.

However, there is an issue with extending applications to cohorts unable to follow specific instructions, such as people with lower education levels. fMRI-neurofeedback requires a high concentration level to learn the experimental procedures and regulate brain activity before and during the neuromodulation session, which may be difficult for many groups of patients (e.g., patients with impaired self-awareness). There may be potential risks, such as inducing maladaptive neural plasticity (e.g., by training in dysfunctional strategies [40]). New approaches are developed to integrate neural feedback obtained from rtfMRI with other neuromodulatory interventions rather than dealing with the complex challenges of dealing with self-regulation as described above while taking advantage of the closed-loop brain modulation approaches adopted in rtfMRI systems to overcome some of these limitations. One of these approaches, namely tES-fMRI, is discussed in the following section.

### fMRI as a therapeutic target biomarker

Recent advancements in biomarker development have shifted the focus from diagnostic/prognostic/predictive biomarkers to the identification of biomarkers that measure “response to treatment” or represent “therapeutic targets”. Response/target biomarkers should be reliable and have a well-established and quantifiable relationship with the clinical outcome of interest in the sense that their change is associated with a change in clinical outcome. There are collective efforts to identify these biomarkers at the group level and validate them at the individual level [41, 42]. Target/response biomarkers would support the development of targeted and individualized treatment protocols by elucidating mechanisms of action in neurologic or psychiatric disorders and offering a personalized neural circuit-based target for behavioral, pharmacological, or neuromodulatory interventions [43].

fMRI (e.g., a certain fMRI functional connectivity patterns/strength) can be a proxy measure for the latent variables (such as changes in network synchronization of neural activity) that causes the change in observable clinical outcomes [44]. Therefore, fMRI can provide a foundation for developing target biomarkers (e.g., fMRI is being used as a target engagement biomarker in drug development for mental health disorders [26]). In this context, a growing number of studies sought to develop fMRI-based biomarkers for targeted neuromodulatory interventions [45–47]. It has been shown that neuromodulation technologies like NIBS achieve their clinical effects by stimulating not only the local brain region underneath the electrode/coil but also distributed brain networks by propagating along related neural circuits [48, 49]. With respect to the distributed effects of NIBS, fMRI has the potential for informing network-level targets for NIBS in the individual. For example, some recent clinical trials have suggested that fMRI-informed NIBS targets derived from individual connectivity may lead to clinical improvements (please refer to this review article [50]). However, most of these studies are based on retrospective analysis or observational trials [22, 51–53]. Prospectively testing the differences between fMRI-informed approaches with conventional methods, and defining an appropriate control condition to quantify the priority of fMRI-informed methods are still lacking.

However, it should be mentioned that targeted neuromodulation using fMRI biomarkers might face several challenges. For instance, (1) despite high response and remission rates in previous fMRI-informed target selection in NIBS research, most of these studies have been performed with small sample sizes or without a well-controlled sham or comparison target group [22, 51–53]. Therefore, larger trials are needed to validate the accuracy and reproducibility of the targeted neuromodulation using fMRI biomarkers. (2) Furthermore, although the idea of using fMRI data (e.g., brain connectivity) to inform neuromodulation is not new, in many previous studies targets were identified using a normative connectome that was derived from an averaged group and not representing individual information [21, 54]. With a growing body of evidence for precision functional mapping studies, there is an effort to move from group-level fMRI target biomarkers to individualized target biomarkers (1). Future studies should focus on how individualized fMRI-informed target selection in NIBS studies is diverging from fMRI-informed methods based on normative connectomes and group-level fMRI data and which one is more useful/pragmatic in clinical applications. (3) Additionally, the instantaneous brain state and its fMRI recording results could be inter-individually variable at the time of stimulation. Defining a target biomarker with respect to ongoing brain function would be challenging. Recent advances in concurrent NIBS with fMRI and designing stimulation protocols that are controlled by ongoing brain function can potentially address this variation [23, 55]. (4) Finally, despite literally tens of thousands of papers published utilizing fMRI measures (either task-fMRI or resting-state) the sensitivity, specificity, and reliability of correlations between imaging findings and symptoms for any diseases at the individual subject level have not yet been established with sufficient rigor to establish fMRI as a clinically useful tool and more research is needed to consider fMRI markers as a therapeutic target in brain stimulation studies.

The development of target biomarkers for neuromodulation can be initiated with previous brain mapping efforts such as fMRI or lesion studies. However, neuromodulation trials can also validate the causal relationship between these neural targets, their proxy measures (e.g., fMRI), and clinical outcomes at the group or individual levels. The process of biomarker development might need to iterate between brain mapping and neuromodulation clinical trials. This process can be facilitated with closed-loop technologies like closed-loop tES-fMRI at an individual level.

### Integrating tES with fMRI

Transcranial electric stimulation (tES) is a safe, portable, inexpensive, and scalable technology that applies low-intensity (≤4 mA) direct (tDCS), rhythmically alternating (tACS), or randomly alternating (tRNS) electrical currents through surface electrodes attached to the scalp to externally modulate brain activity [56]. tES induces minor shifts of neural membrane potentials that are too weak to sufficiently depolarize cortical neurons to induce action potentials [57], but alter spontaneous brain activity [58, 59]. tES is used in a wide variety of applications in health and disease to change the excitability of the targeted brain area/network both acutely during the stimulation as well as transiently outlasting the stimulation via plasticity-inducing protocols [56, 60, 61]. Despite promising results in a growing number of tES studies, some significant limitations have become increasingly apparent over the years, and many questions remain unanswered regarding the neural basis of tES effects, as well as strategies for their optimization, taking inter- and intra-individual variability into account [59, 62–64].

One of the greatest challenges in the application of tES is to understand how this stimulation can be individualized and targeted more precisely to efficiently interact with the ongoing brain state in each individual [64]. Utilizing fMRI to measure how tES modulates brain targets seems promising [65] and has received much attention in recent years [66], providing new concepts of how tES interacts with functional brain activity not only in targeted brain regions but also at the individual whole-brain level [65, 67]. Studies have focused on the role of the initial brain state, the immediate effects during the application of tES as well as its subsequent after-effects. Accordingly, tES-fMRI can be performed in a consecutive approach where imaging data are collected before and/or after tES intervention (offline tES-fMRI) or in a concurrent approach where stimulation and imaging data acquisition are performed simultaneously (online tES-fMRI)) [68]. Concurrent tES-fMRI enables researchers to engage brain targets while monitoring the ongoing brain state directly. However, tES inside the scanner in consecutive or concurrent approaches requires specific practical considerations [69]. A checklist was recently developed for concurrent tES-fMRI obtained from a Delphi consensus process regarding essential factors that should be checked during a concurrent tES-fMRI trial to ensure transparency, completeness of the report and to improve study replicability and general reporting practice of concurrent tES-fMRI trials [70].

Although implicit rtfMRI-neurofeedback, in which both feedback and the instructions are orthogonalized, helps to reduce some issues with the correlation between instructions and target brain state, the main advantage of tES-fMRI trials compared to rtfMRI-neurofeedback studies with an explicit strategy which is commonly used in clinical applications (where subjects learn to associate the presented feedback signal with their brain activity and learn to volitionally control it), is removing the effects that depend on instructions and learning. However, titrating stimulation dose for modulating brain functions is complex as the impact of tES is highly dependent on the stimulation parameters and ongoing brain activity. Real-time tES-fMRI, in combination with an optimized closed-loop control, will help to measure and verify the changes in brain functions targeted and induced by tES.

### Closing the loop between the brain and the stimulator

To date, the vast majority of tES-fMRI studies have employed open-loop brain state-independent approaches, in which stimulation is applied with constant parameters independent from brain state and respective dynamic alterations. This one-size-fits-all approach ignores inter- and intra-individual variabilities, which might contribute to mixed results in experimental and clinical trials [71]. Nowadays, it is, however, technically possible to close the loop between the measured output of the brain and the stimulator [11, 12, 55, 68, 72–74], allowing to take advantage of closed-loop brain modulation approaches used in rtfMRI-neurofeedback studies (Sulzer et al. 2013) and thus adapt stimulation parameters to ongoing fluctuations in brain physiology dynamically.

**Fig. 2:**
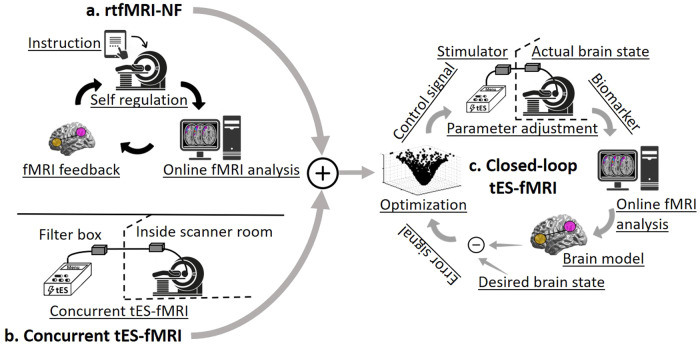
tES-fMRI and real-time fMRI-neurofeedback integration into a closed-loop system to optimize dose titration in tES studies. **a** Real-time fMRI-neurofeedback system (rtfMRI-NF). In rtfMRI-NF interventions, subjects are asked to regulate their brain functions based on an instruction to engage specific neural targets. BOLD signals are analyzed with a rapid algorithm in each loop, and the level of target engagement is visualized. Subjects regulate their brain activities based on the instruction to maximally activate predefined neural targets. **b** Concurrent tES-fMRI system. The tES stimulator is placed outside of the MR scanner to avoid associated noise, and fMRI data are collected simultaneously with tES to measure the modulation of the neural targets. **c** Closed-loop tES-fMRI. In a closed-loop tES-fMRI setting, the fMRI data are acquired and analyzed in a real-time approach in response to the stimulation to measure and reach the ideal target with optimized stimulation parameters. rtfMRI real-time functional magnetic resonance imaging, NF neurofeedback, tES transcranial electrical stimulation.

Using BOLD signals to model the current brain state and continuously fine-tune tES parameters based on differences between the measured brain state and desired brain state would close the loop between the stimulator and the brain. It is expected that the closed-loop tES-fMRI approach is thereby able to induce the intended modulation of functional activity, which can be measured by the target engagement biomarker, as indexed by rtfMRI data, in an individualized manner. This external adaptive stimulation and automatic controller would overcome the limitations of subjective responsiveness to instructions and self-regulation in conventional fMRI-neurofeedback studies and inter-individual variability in tES trials discussed above. In a real-time closed-loop tES-fMRI system, the neural feedback is provided to the optimization protocol controlling tES parameters instead of delivering it to a human subject as in conventional neurofeedback protocols (Fig. 2).

The closed-loop tES parameter optimization approach suggested by Lorenz and colleagues [75] could be an effective approach to reduce uncertainty about appropriate neurostimulation parameters. In a closed-loop tES-fMRI system, differences between the brain model and predefined targeted brain activity obtained from the concurrent fMRI biomarker are used iteratively to adjust stimulation parameters (e.g., phase difference, frequency, intensity, or any other spatial or temporal variable). Stimulation parameters optimally suited to reduce deviations from the predefined brain state (ideal value) are determined. This optimization can be performed immediately after receiving the first brain state measurement, and every stimulation parameter is then updated based on the performance of the previous one. An optimization algorithm can be run based on multiple feedback cycles. After testing several stimulation parameters in an optimization algorithm, the best-performing parameters will be used for the next round of stimulation. This iterative process for adjusting the stimulation protocol can be continued until tES achieves and maintains the desired brain state, or no further improvement can be accomplished.

There are a few attempts to close the loop between tES and brain states using EEG to estimate the current brain state, because of its cost-effectiveness and portability and its high temporal resolution, which allows extracting information in real-time without considerable inherent delay of brain dynamics at faster time scales, i.e., neuronal oscillations, that are often the target of entrainment approaches via tES [12, 68, 76, 77]. For example, EEG-based phase-locked tACS was used to stimulate brain targets relative to ongoing brain oscillations, and autoregressive (AR) modeling was used to predict the future EEG signal from a segment of the past EEG in order to adjust stimulation parameters [78]. In this implementation, stimulation was triggered to occur within specified time intervals (e.g., synchrony with online detected brain oscillations based on a specific power threshold). However, accurate forward modeling of all EEG components is challenging and time-consuming under real-time constraints. In other studies, an EEG-feedback-controlled approach was used to restrict the application of tACS at the respective frequency with phase alignment to the occurrence of sleep spindles [79, 80] or slow oscillations [19, 81, 82]. A case study with a closed-loop tACS-EEG system reported that stimulation in the gamma frequency band suppressed endogenous alpha oscillations since these two frequencies are acting antagonistically [83]. Conversely, in a recent semi-closed-loop (with an open-loop state-dependent approach [12]) tACS-EEG study, it was reported that alpha-band frequency suppressed gamma-band brain oscillations [84].

One of the main challenges for concurrent tES-EEG (specifically tACS-EEG) is the high-amplitude EEG artifact. The magnitude of artifacts induced by tES is several orders larger than the magnitude of the neuronal signal in the EEG, and a truly closed-loop tES-EEG approach would require the online removal of such artifacts and noise from the recorded EEG signal in real time. Although EEG is a cheap portable device that can be integrated with tES equipment to record neural activity with a suitable temporal resolution (unlike fMRI), there are inherent physiological artifacts during concurrent (online) tES-EEG setups [85].

Different groups suggested sophisticated experimental designs or demanding real-time computational procedures to remove this artifact, imposing additional restrictions that are not readily adaptable for clinical applications. Although there have been some promising attempts to overcome this barrier [86, 87], there are still many challenges to a successful recording of actual cortical activity during the concurrent application of tES. Several artifacts are emerging from the interaction of physiological activity and tES as well [85]. In case of successful entrainment, the spatial and temporal properties of the electric current originating from the stimulation (artifacts/noise) and those originating from neuronal activity (true signal) are perfectly correlated in time and space, thus making the successful removal of respective artifacts computationally at least very demanding, if not impossible, and it would be very difficult to derive EEG/MEG measures of target engagement in a real-time protocol [88, 89]. This limitation keeps closed-loop tES-fMRI as one of the main competitors in closed-loop NIBS neuroimaging despite its cost, size, and lower temporal resolution.

### Closed-loop tES-fMRI setup

**Fig. 3:**
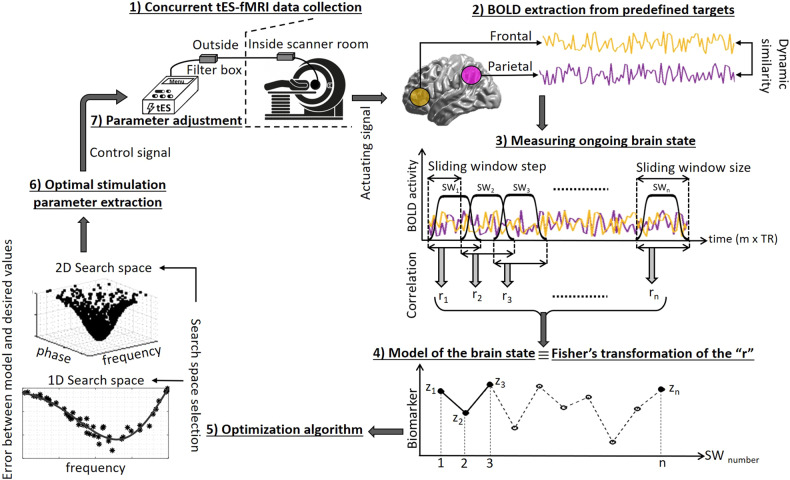
The process of integrating tES with fMRI in a real-time closed-loop approach (Closed-loop tES-fMRI). (1) Concurrent tES-fMRI starts with prior expectations about optimal tES parameters. (2) Targets are selected based on the clinical/behavioral outcome of interest and its corresponding neurocognitive function. The averaged BOLD signals are extracted from predefined targets. (3) To measure ongoing brain state (e.g., frontoparietal connectivity), the BOLD signal is segmented using a tapered sliding window, and dynamic similarities between extracted BOLD signals in frontoparietal regions of interests (ROIs) are calculated for each segment using the Pearson correlation coefficient (*r*). (4) Fisher’s Z transformation of those correlation coefficients is used to measure dynamic functional connectivity (FC), i.e., the dynamic correlation between the time series of frontal and parietal ROIs is defined as a model of current brain state over time. (5) The extracted measures are compared with the desired value and the results of the respective comparison are fed into an optimization algorithm. (6) Optimal stimulation parameters are determined to minimize the difference between ongoing FC and the desired value by maximizing the objective function in a defined parameter search space (e.g., 1D search space to optimize the frequency of the injected current or 2D search space to optimize phase difference and frequency simultaneously). (7) The stimulation device is updated with the optimal stimulation parameters for the next round, and this loop continues until predefined stopping criteria are reached. tES transcranial electrical stimulation, fMRI functional magnetic resonance imaging, BOLD blood oxygenation level-dependent.

With respect to the limitations of closed-loop tES-EEG systems and benefits of concurrent tES-fMRI, there are potentials for a pipeline integrating tES with fMRI in an individualized approach by optimizing stimulation parameters based on ongoing brain states (fMRI biomarkers such as activity/connectivity or multivariate patterns). Because typical signal processing methods cannot reliably remove physiological artifacts from the recorded online tES-EEG signals and more consideration should be taken into account to denoising online tES-EEG stimulation artifacts, here, we only focus on online tES-fMRI systems. Fig. 3 visualizes this pipeline and shows an example of a real-time closed-loop tES-fMRI system.

In the first step, tES as an intervention will be applied concurrently with fMRI data acquisition in order to modulate a specific brain target. Two simple approaches can be suggested; (1) maximizing activity or connectivity: the region of interest or connectivity between regions is determined based on a predefined hypothesis; the stimulation parameters are then optimized to maximize/minimize the activation of the region of interest or network connectivity, (2) maximizing similarity to a desired map: in addition to optimizing region of interest activation or connectivity, it is also possible to minimize the differences between current brain state and a predefined desired map. Here the provided feedback does not provide the achieved activation or connectivity change, but the likelihood between the predetermined functional map and current brain state is fed into the controller. The current brain state obtained in each iteration is compared with the desired map, then the controller receives the error signal and the main goal of the optimization algorithm is therefore to adjust the stimulation parameters to align with the predetermined brain state. A desired functional map can be defined based on the activation maps produced in previous fMRI studies, functional/anatomical atlases, or individual-level approaches (e.g., functional localizer, individual-level parcellations).

To provide an example of the first approach, we have selected frontoparietal connectivity within the executive control network as the target that should be modulated by the injected current. The average time series of the voxels within the nodes of interest (e.g., spheres in frontal and parietal cortices) are extracted online. Next, for modeling the brain, the dynamic functional connectivity between the time series is calculated using the Pearson correlation coefficient (*r*) in a sliding window (e.g., with a step size of one TR), followed by Fisher’s Z transformation to normalize the highly skewed distribution of values. The “*z*” values are determined for the time series within the sliding window in each step to generate a measure suited for brain state modeling (frontoparietal connectivity) over time. The optimization algorithm (as a controller) tunes stimulation parameters based on a systematic search procedure through a defined parameter space to maximize frontoparietal connectivity. Finally, based on the control signal command, the stimulation parameters will be updated for the next round of stimulation.

This paradigm should be tested experimentally to identify potential confounds of the design. We previously performed a systematic review to propose a checklist for concurrent tES-fMRI experiments [70]. We updated our systematic review by the end of April 2022, and we found that of all currently available tES-fMRI studies, none has used a closed-loop brain stimulation protocol. Recently, in our team, we tested an online closed-loop real-time protocol, and the initial feasibility and applicability of this system were investigated in a pilot study [23]. In that study, we delineated how a closed-loop tES-fMRI study aiming for frontoparietal network modulation can be designed and performed. We discussed challenges related to artifacts, the temperature of the electrodes, and the online optimization algorithm. The initial results provide reasonable evidence for the safety, noise control, and feasibility of closed-loop tES-fMRI in two participants [23]. Due to the inception phase of closed-loop tES-fMRI, there are several challenges to be overcome to make this an effective treatment option for clinical trials, while the field is principally important, and might result in major advances of this intervention in the future.

### Challenges of closed-loop tES-fMRI systems

**Fig. 4:**
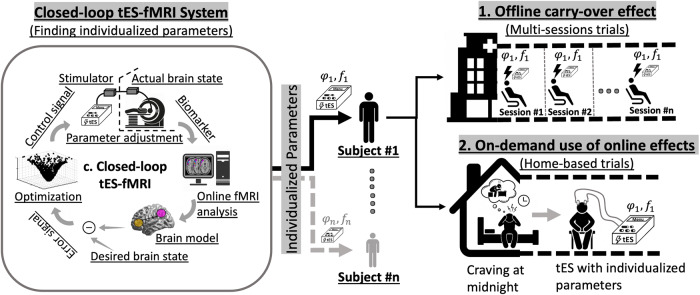
From closed-loop individualization to real-world clinical application. The clinical utility of optimized parameters obtained from closed-loop tES-fMRI can be tested in two main types of subsequent trials. (1) Multi-session trials to test whether the online effect will be transferred as accumulating and long-lasting offline effect meaningful in the clinical setting and (2) Trials with home-based on-demand use (e.g., when a person with substance use disorders feels a high level of craving at midnight) of the online effects (e.g., momentary reduction in craving during stimulation) will have a meaningful effect on clinical outcomes (e.g., relapse or overdose prevention).

The main technological and methodological challenges for closed-loop tES-fMRI include:

1. Timing: Based on the proposed pipeline in Fig. 3, in order to detect stimulation effects on neural targets, this effect should be large enough in the time scale of the sliding window to be detectable. Therefore, window duration and slide duration should be selected based on the temporal resolution of the dynamic response observed in the targeted brain regions. Furthermore, the amount of data that should be collected (duration of data collection) is related to the stimulation effect; the smaller the effect, the more data will be needed. Moreover, the duration of the wash-out period between iterations required to avoid carry-over effects makes the situation considerably more complicated.
2. Temporal delay due to HRF delay: Although fMRI tools have been used as a marker of brain state in concurrent tES-fMRI studies [90–92], the specific temporal characteristics of the BOLD response to induced electrical stimulation remain unclear [93]. The time lag of the hemodynamic response induces a temporal delay in feedback delivery in closed-loop systems. Since the BOLD signal is slow and delayed relative to the neural activity it reflects, this time delay should be considered in defining the sliding window starting time in the optimization algorithm as defined in Fig. 3 and suggested by [23].
3. Computational delay: A delay in acquisition and computation of the feedback signal is added to the intrinsic hemodynamic response in closed-loop rtfMRI systems. However, recently published papers for analyzing rtfMRI data revealed that all fMRI processes with extensive real-time denoising can be performed in a few seconds or even less than 1 sec to keep the pace of real-time data processing sufficient to avoid the accumulation of a relevant delay [94].
4. Immediate brain responses to stimulation vs. aftereffects: One important issue in designing a tES-fMRI trial is the mechanistic timeline of interest. The effects of tES on cortical excitability can be separated into immediate (online) brain response vs. after-effects (offline), e.g., lasting changes in synaptic efficacy. These effects could be targeted and recorded based on the temporal order of stimulation and neuroimaging; concurrent vs. consecutive stimulation and recording. Online stimulation protocols try to measure the immediate effects of tES via a concurrent imaging paradigm while offline protocols with subsequent imaging aim to measure aftereffects. Offline and online effects could provide different yet relevant information on brain processes and cognitive functions in response to the stimulation [95]. Online effects take advantage of real-time monitoring of the brain state while stimulation induces changes in brain activity. This may help to increase the precision of the stimulation based on the effects of the stimulation (e.g., stimulation modulates online motor learning [96, 97]). However, with respect to the state dependency of tES, the level of background neural activation during the application of tES affects the stimulation outcomes. As tES does not directly evoke action potentials but modulates spontaneous cortical activity, which depends also on dynamic brain state, quantifying the online effects of tES is complex and may make it difficult to show concurrent changes in BOLD signals. Offline protocols involve neuronal activity alterations that continue beyond the stimulation and are labeled aftereffects. Although the offline method reduces the level of complexity, it may increase the variability in response to tES because it can abolish dynamics of brain state alterations caused by temporally increasing effects of ongoing stimulation. Concurrent alterations of the target engagement dynamics cannot be tested with offline tES-fMRI trials while dynamic changes might not be a problem, but a factor that can be taken into account with online tES-fMRI approaches [98].
  Both online (concurrent effects) and offline (immediate aftereffects) protocols could be used to close the loop between the brain and stimulation parameters [68]. In the online closed-loop system, stimulation parameters are iteratively and automatically optimized inside the scanner based on the ongoing brain state during the application of tES while in the offline closed-loop system, immediate brain responses (after the end of stimulation) are used to update stimulation parameter space. However, closing the loop in offline approaches might be difficult because the brain should be stimulated again and the respective stimulation-free interval might cause nonlinear dynamics while in online stimulation parameters could be changed in a closed-loop manner which will be advantageous [98]. Closed-loop systems compared to one-size-fits-all open-loop systems in general offer enhanced parameter selection based on adapting the stimulation parameters to the brain function dynamics in an online or offline manner and allow us to adjust stimulation parameters based on brain function which can be clinically valuable by itself (e.g., a personalized tES headset can be used with the optimized stimulation parameters to reduce momentary craving or suicidal ideations that a patient can experience anytime during the day).
  Online methods trace underlying brain functions and provide real-time triggering of tES based on the ongoing intrinsically generated brain activity. Therefore, online closed-loop systems are useful if the changes in the dynamics of the brain state of interest have a slow frequency that could be detected by the BOLD signal with limited temporal resolution. The online closed-loop system is also clinically valuable. It considers the patient’s status (e.g., brain functions during experiencing craving while being exposed to drug cues), adopts stimulation parameters to the current brain state, and could be capable of observing responses to the adaptive stimulation in each iteration. One possible scenario is that optimized stimulation parameters will help to engage the desired brain state. The clinically desired brain state which is engaged by the optimized stimulation parameters will hopefully stay as a long-lasting aftereffect (as Nitsche and Paulus showed that the directionality of the online and aftereffects are identical [99]) and will accumulate with multiple sessions of the optimized tES. To the best of our knowledge, so far, there is only one actual closed-loop tES-fMRI study available [23]. That single successful trial used rapid online feedback and iterative evaluation of immediate tES effects without any report of the long-lasting offline effects. Multiple ongoing and future closed-loop tES-fMRI trials will bring more empirical evidence to address this challenge.
5. Task-based fMRI design: In task-based data collection, the task that participants perform during stimulation sessions can change brain states, and consequently stimulation effects [100]. Therefore, for parameter optimization to increase the efficacy of tES, task selection is critical. Task design should activate brain regions related to the desired brain state one wishes to target. Fatigue or learning effects, including adaptation or habituation that can cause a significant decrease in the BOLD response, should be minimized during task performance [101–104]. Optimization of task parameters can also be performed in a closed-loop manner, as suggested by Lorenz et al. to maximize the similarity between ongoing brain activity and a target brain state [105]. It should be noted that, in task-based paradigms, further considerations are required regarding the simultaneous effects of the task and tES. Separation of stimulation and task effects is nontrivial—alterations can be caused by tES directly or indirectly by tES-induced changes in task performance. Indeed, task performance can be also used as an outcome measure in closing the loop. However, the focus of this paper is on closing the loop based on brain activity, not behavior.
6. Resting-state fMRI design: Resting-state fMRI can also be used to model the current brain state. However, since tES does not directly induce action potentials, in each iteration of a closed-loop system, the online changes of tES might be more challenging to quantify during rest compared to task performance [68]. During resting-state fMRI (especially BOLD fMRI), the BOLD response seems to be weaker compared to tES-modulated task-evoked BOLD responses. With respect to the innate fluctuations in resting-state fMRI and lack of consistent signals especially during short periods of data recording in each iteration of the closed-loop system, detecting brain state changes during task-free real-time BOLD-fMRI for optimizing stimulation parameters would be complicated. Extra caution must be taken in designing robust and sensitive fMRI experiments to capture online tES effects.
7. Peripheral tES mechanisms: sensory modulation (e.g., cranial nerves, retina, vestibular organ, trigeminal nerve) can also induce effects on brain activity due to conscious perception or subconscious processing of peripheral input to the brain and may open the possibility of an indirect brain modulation via peripheral input. Therefore, the simultaneous stimulation of sensory organs or cranial nerves can complicate the optimization algorithm to interpret brain activity alterations in response to stimulation. This complexity is a general issue in transcranial stimulation protocols not restricted to closed-loop systems or tES. However, it might affect the interpretation of the optimization results, and thus considering these effects is relevant. Although more studies are needed to clarify to what extent tES effects are mediated by transcranial pathways or other mechanisms of action, this peripheral stimulation effect is however likely not the primary driver of tES responses [106, 107].
8. Inherent brain oscillations: The adaptation of fluctuations, especially concerning the phase of spontaneous activity, is challenging with this approach due to the limitations of the sampling rate, which restricts the investigation to slower oscillations with BOLD signals. Phase, frequency, and amplitude of neuronal oscillations can change local or global brain states, altering both the immediate brain response and subsequent after-effects of the tES intervention. For a better understanding of the possible mechanism of tES in changing brain oscillations, considering in-vivo animal studies may also be helpful [108–110]. For example, single unit recordings in nonhuman primates revealed that tES (specifically tACS) affects spike-timing in a dose-dependent manner such that via increasing the extracellular electric field strength more neurons are entrained to the stimulation frequency [111]. Spike-timing-dependent entrainment changes underlying brain oscillations that can inform us about communication between brain regions [112, 113]. Additionally, integration of EEG and fMRI in a hybrid simultaneous acquisition (that is however technically demanding) can potentially inform about the relation between BOLD signals and specific oscillations.
9. Response variation: Trial-to-trial variability (test-retest) needs to be smaller than the effect of interest (effects of stimulation parameters on brain function). Measurement errors related to small effect sizes can affect optimization algorithms [114]. Therefore, to assess the efficacy of closed-loop tES-fMRI at the individual level, a power analysis should be conducted to ensure adequate power of the chosen experimental protocol to measure a given effect size, e.g., by considering a sufficient number of image recordings per optimization iteration or aggregating data from multiple rounds, or a sufficient number of iterations to identify optimal parameters.
10. Signal-to-noise ratio (SNR): Interferences between stimulation and the imaging magnet coil might cause false-positive detections and reduce the SNR, which would make it difficult to record the neural target of interest accurately. With respect to advancements in MRI technology and better RF shielding of the cables that enter the MRI scanner room, although a reduction of the absolute MR signal due to the presence of the tES electrode (reduction of signal intensity of 11%) was reported [115], however, no significant effect of tES electrodes on the SNR of the underlying brain regions was detected [116].
11. Brain state measurement: The potential of fMRI-derived measures to model current brain state draws increased attention [117–121]. However, finding a reliable, sensitive, and accurate measurement for closing the feedback loop in a real-time tES-fMRI system is challenging. This would require a state-dependent fMRI signal that is (1) accurately and reproducibly measured over time, (2) affected by the induced current, (3) predicting stimulation outcomes, (4) rapidly reflecting alterations in the targeted brain area (modulation of the target) by iteratively optimized stimulation parameters, and (5) reflecting performance that is clinically relevant, which is not trivial.
12. Parameter search space: Two important questions should be answered in selecting the stimulation parameter search space. (1) “Which parameters should be optimized?” Different parameters can be optimized during the application of tES, such as intensity, frequency, and phase difference of the injected current, selection of the stimulating electrodes, or stimulation duration. Selecting the parameters to be optimized can be complicated [105]. (2) “How large should the search space for each parameter be?” The possible range (based on the safety and tolerability of the intervention) is too large to be screened for each parameter, e.g., 0.1 Hz to kHz for frequency, 0-359 degrees for phase, and 0.5–4 mA for current intensity. Exploring all possibilities results in lengthy and impractical testing procedures [75]. With previous results at hand, the search space can be limited to fine-tuning within a reasonable area. In a pilot database, search space can be constrained around a predefined target value. However, a huge search space is unavoidable when no prior information about conditions can be leveraged from previously published studies or meta-analyses.
13. Optimization algorithm: Finding an efficient algorithm to adjust stimulation parameters based on online biomarker recording to find the global optimum in a reasonable time is challenging. Optimization algorithms should be robust, fast, and easy to implement in a real-time system. Previous brain stimulation studies have shown that Bayesian optimization can be a useful tool for optimizing stimulation parameters [75, 122, 123]. However, it should be noted that despite the effectiveness of Bayesian optimization in a previous case study [122], the amount of computation required for generating candidate parameters in a real-time approach might be prohibitively resource-demanding. Other optimization methods such as the simplex algorithm [124] or convex optimization [125] methods might be candidates for a closed-loop system with a big search space. More investigations and empirical evidence are needed to determine an efficient algorithm that takes little time to evaluate candidate solutions.
14. Validation: It would be essential to test whether optimal stimulation parameters indeed have an impact on clinical outcomes. Similar to other clinical interventions, for closed-loop tES-fMRI, a systematic, empirically based approach is needed for assessing the effectiveness and utility of the optimization in clinical trials. Defining neurophysiological, behavioral, or cognitive metrics following closed-loop tES-fMRI is necessary for evaluating levels of efficacy to provide a foundation for the evaluation of closed-loop tES-fMRI and its application in clinical neuroscience research. However, any change induced by a closed-loop tES-fMRI system would be difficult to measure because (1) any effect will take place on potentially moving targets (dynamically changing brain states might be caused not only by actual stimulation but also be triggered by previous stimulation iterations or resulting from intrinsic changes in functional activity), [2) creating a double-blind condition for a concurrent tES-fMRI and maintaining a blinded condition would be critical in cross-over designs when participants receive both active and control interventions in the same session. Control conditions can be defined in different ways, e.g., sham tES parameters, active tES without optimized stimulation parameters, or sending feedback to the controller that does not represent the actual current brain state. Rigorous designs are required to explore the best control conditions in randomized clinical trials based on the research question.
15. fMRI-based target selection for translational psychiatry: While not utilized in clinical psychiatry at present, closed-loop NIBS protocols have the potential to modulate impaired activity-dependent neural circuits (e.g., closed-loop TMS-EEG setup for guiding TMS with brain activity feedback [20]). However, as suggested by Taylor et al., when the study is focused on disease correlates (e.g., drug craving or consumption), the causal interpretation of fMRI findings would be ambiguous and the correct interpretation of these correlates (e.g., causation, compensation, or coincident relation) is essential for developing effective neuromodulation targets [126]. Using real-time closed-loop tES-fMRI protocols as a therapeutic option for psychiatric disorders by targeting their neural circuitries will need to pass multiple levels of experimentation. Disease outcomes might not necessarily improve after normalizing abnormal neural circuits (e.g., by modulating with an electrical current) associated with that disease (e.g., an fMRI biomarker may change as desired in response to stimulation, but this may not reflect the desired change in the disease-relevant underlying circuits).
16. Experimental trials should validate the causal relationship between the target (biomarker) engagement and disease-specific neural circuits and behavior (clinical outcome of interest) using closed-loop tES-fMRI protocols.
17. Endurability and clinical considerations: In the context of brain-state-dependent stimulation, closed-loop systems iteratively change stimulation parameters via a control signal to reach and maintain a predefined brain state based on tracing a certain brain-state biomarker and reducing deviation from the desired state (i.e., reducing the error signal). The stimulation should be strong enough to enact clinical change and endure to be meaningful in most clinical applications. As we discussed above, in certain clinical applications online effects even without lasting offline effects could be substantially important, e.g., immediate interruption of intrusive thoughts or feelings such as suicidal/homicidal thoughts or drug cravings to prevent immediate subsequent behaviors like suicide or drug use/relapse. In these scenarios (Fig. 4), after finding the optimum tES protocol using a closed-loop tES-fMRI data collection, the resultant optimum individualized parameters (e.g., stimulation intensity, phase difference, stimulation frequency) could be applied for each person over consecutive multiple offline tES sessions or as needed with home-based devices for clinical utility in real-world applications. There are recent advances in the feasibility of individualized portable or home-based tES [50, 127–129], but the clinical applicability of online personalized neuromodulation followed by home-based tES using the identified personalized protocol should be tested in future trials.

Based on the currently available empirical evidence for closed-loop tES-fMRI, it is still empirically unclear whether an online closed-loop approach will (a) cause lasting changes beyond the immediate change during the application, and (b) if so, whether these are strong enough to be clinically relevant. However, there is accumulating evidence for both the endurability and clinical significance of tES (non-optimized with closed-loop fMRI) [130, 131]. The durability of the effects of closed-loop tES-fMRI could be discussed based on the engagement of synaptic plasticity as a putative mechanism to mediate outlasting effects via modulating the membrane potential of neurons which has been reported in in-vitro tES studies [132]. It has been shown that network activity could affect synaptic efficacy through a mechanism which is named spike-timing-dependent plasticity (STDP) [133]. STDP suggests that tES leads to synaptic changes based on the modulation of neuronal firing rate in the targeted neural network [134]. As an example, Schwab et al. reported that STDP can explain stimulation-outlasting connectivity modulation induced by dual-site tACS [135].

Furthermore, there is supporting evidence for using a closed-loop strategy that dynamically updates stimulation parameters in response to ongoing brain responses in other types of brain stimulation methods like deep brain stimulation (DBS) that showed long-lasting treatment effects [136]. Basically, all neurofeedback approaches are also based on the idea of changes in synaptic plasticity and thus aftereffects being caused by the feedback [137]. Additionally, in non-closed-loop systems, the effects of increasing the duration of applying tES on long-lasting effects like early and late long-term potentiation (alterations lasting for more than 3 hours) have been investigated [138]. In some studies, periodical stimulation with an interval of up to 24 hours showed enhanced efficacy of the second stimulation that suggested a cumulative effect of spaced stimulation which shared similarities with late LTP processes [139] such that weak tES caused a relatively strong cumulative effect of synaptic plasticity [140]. Furthermore, multi-session tES has produced more robust gains in stimulation outcomes like motor skill learning that could persist for at least 3 months after the intervention [131]. A recently published closed-loop tES study protocol also used three sessions of closed-loop tES-EEG to provide patient-tailored treatment for people in a minimally conscious state [141]. However, it is worth mentioning that, even by achieving an optimal subset of stimulation parameters, there is no guarantee that clinical outcomes will last long enough to be clinically meaningful and the clinical relevance is indeed an open empirical question. Future research will tell us whether a closed-loop tES-neuroimaging technique system is worth the effort.

### Potentials of closed-loop tES-fMRI systems

Despite the above-mentioned challenges for developing a closed-loop tES-fMRI system, this method has a unique set of potentials covering a wide range of applications, from an advanced neuroscience research tool to a precision medicine treatment for psychiatric and neurologic disorders. Some benefits of closed-loop tES-fMRI are categorized below.

1. Objective optimization: A closed-loop tES-fMRI system does not require participant training for subjective control over the brain (as rtfMRI-neurofeedback requires). The optimization algorithm automatically adapts stimulation parameters (as suggested by Lorenz et al. [105]) based on the measured data signal. Participants thus are only asked to engage in a task or resting state. This algorithm-based objective optimization eliminates learning and instruction biases.
2. Context-dependent preferences: tES may preferentially modulate brain regions/networks that are already activated, for example, by a specific task [108, 142], the task-modulated regions/networks can differentially benefit from the induced EFs [59, 100]. Iterative measurement of the brain state in a closed-loop tES-fMRI system can help to optimize stimulation parameters based on the specificity of tES, and stimulation parameters will be updated based on the concurrent context/task (brain function of interest).
3. Individualized parameter optimization: Using optimal parameters based on individualized biomarkers may help to boost the electrophysiological and behavioral effects of the stimulation [143], reduce inter-individual variability of tES response, and offer the potential for an individualized approach for identification of optimal parameters (e.g., improving tES efficacy by application of stimulation with the individual dominant frequency [144–146], phase [147, 148], or electrode placement [149] according to individual functional maps, as reported in previous studies).
4. Availability of multiple measurements: fMRI analysis can cover the whole brain and detect and process various biomarkers for modeling the current brain state in response to the applied stimulation (e.g., connectivity between DLPFC and insula and DLPFC and IPL for top-down regulation and frontoparietal interactions with respect to tES applied over the DLPFC). Multiple measures can be fed into the optimization algorithms, which can potentially increase the robustness of closed-loop tES. Information about the current brain state originating from diverse sources might be helpful whenever an error (e.g., low SNR) occurs in one extracted measure. In this situation, there will be another relevant measure to steer the closed-loop optimization procedure and decrease the probability of inaccurate adjustment of stimulation parameters. This potential of multivariate biomarkers should be further explored not only in tES-fMRI but also in other areas of neuroscience.
5. Individualized treatments/precision medicine: Previous studies provide exciting proofs-of-concept of how personalized brain stimulation might benefit clinical trials [146, 150–152]. Understanding the therapeutic susceptibility to NIBS via data obtained from functional imaging, such as fMRI measures in closed-loop tES-fMRI systems, will help to develop patient-tailored tES strategies toward precision medicine and personalized nonpharmacological therapy [153]. The optimized parameters that are defined for each individual can be potentially used outside the scanner and even in home use settings through portable tES devices [154]. The success of closed-loop tES-fMRI in clinical applications will largely depend on the development of brain-based biomarkers to translate the observed transient stimulation effects to a sustainable recovery in patients. Long-term longitudinal studies are still needed to validate respective assumptions [155].

### Closed-loop tES-fMRI in translational psychiatry

fMRI is commonly used as a noninvasive neuroimaging technique to explore the neuropathological mechanism of different psychiatric disorders [156]. fMRI studies showed that psychiatric disorders alter multiple functional networks compared to typically developing individuals [157, 158]. Hundreds of studies reported different levels of relationships between behavioral and fMRI measures in psychiatric disorders trying to introduce targets for neuromodulatory interventions [159, 160]. Our recent systematic review revealed that up to November 2022, 275 tES-fMRI studies/trials were published (92 concurrent tES-fMRI trials) [66]. 189 tES-fMRI studies recruited healthy participants while multiple tES-fMRI studies targeted psychiatric disorders including schizophrenia (*n* = 8), depression (*n* = 6), bipolar disorder (*n* = 1), methamphetamine use disorders (*n* = 4), nicotine use disorder (*n* = 3), alcohol use disorder (*n* = 1), gambling disorder (*n* = 1), ADHD (*n* = 1), fibromyalgia (*n* = 2), high trait anxiety (*n* = 1), posttraumatic stress disorder (*n* = 1), subjective cognitive decline (*n* = 1), and mild cognitive impairments (*n* = 5). For instance, a randomized double-blind sham-controlled trial with 30 minutes of tDCS over the prefrontal cortex among individuals with schizophrenia showed a significant correlation between improvement in working memory performance and increased activation in the medial frontal cortex beneath the anode in the active compared to sham groups [44]. Improvement of executive functions in the active group was associated with reduced activity in the anterior cingulate cortex and offered a potentially novel approach to altering frontal cortical activity and exerting pro-cognitive effects in schizophrenia [161]. In another study, the application of anodal tDCS to the left inferior frontal cortex in a group of participants with mild cognitive impairment exerted beneficial effects on cognition and brain functions [162]. In that study, tDCS effects resulted in the normalization of abnormal network configuration during resting state and improvement in task performance [26]. Application of 20 minutes of tDCS over the DLPFC in a group of participants with methamphetamine use disorders also showed a significant reduction in cue-induced craving and this reduction was correlated with tDCS-induced alterations in both resting-state and task-based functional connectivity in large-scale brain networks [163, 164].

However, despite promising results obtained from open-loop/non-optimized tES-fMRI paradigms, randomized clinical trials showed inconsistent therapeutic outcomes with large inter-individual variations, and clinical trials showed heterogeneous results across participants with the same intervention protocols [65]. More personalized treatment options with patient-specific setups can potentially maximize the clinical efficacy of brain stimulation methods and move psychiatric neuromodulation forward. Closed-loop tES-EEG trials also showed promising results (e.g., proof-of-concept studies like tDCS-EEG in a minimally conscious state [165], and epilepsy [166, 167]) that added clinical value to closed-loop tES trials like closed-loop tES-fMRI.

The promise of closed-loop tES to provide precision medicine tools is also important for establishing noninvasive brain stimulation methods as a viable treatment for psychiatric symptoms [74]. In this regard, future closed-loop tES-fMRI protocols have the potential to open an innovative path in the development of personalized treatments in psychiatry. fMRI has been applied as a tool for monitoring the concurrent effects of tES, with reasonable accuracy and reliability [70]. Meanwhile, fMRI signals have been considered sufficiently reliable and stable in clinical applications (e.g., routinely used in presurgical mapping, and localization of brain functions [22, 168]) when acquired and processed adequately. Translational psychiatry with tES and fMRI is a rapidly expanding field and at least two directions should be considered for the application of closed-loop tES-fMRI for psychiatry; (1) translating a clinical concept into physiological signals that are “excitable” and “measurable” in closed-loop tES-fMRI methods and, the other way around, (2) translating physiological effects of closed-loop tES-fMRI systems into clinical applications. The first direction (from clinics to closed-loop stimulation) would lead to a better understanding of the neural substrate of a disorder while the other direction (from closed-loop stimulation to clinical praxis) would be focused on the benefits for patients and precision medicine. A recently cleared TMS protocol suggests fMRI as a new gold standard for individualized TMS that may play an important role in future clinical trials [169]. SAINT, the FDA-cleared neuromodulation system for major depressive disorder (MDD) uses fMRI data to accurately determine the individual stimulation target for DLPFC modulation. This fMRI-informed TMS was effective in treating 78.6% of patients with MDD. This fMRI-based approach may furthermore offer new insights for individualized tES interventions. fMRI brain-state triggered closed-loop NIBS setups have thus potential to enable psychiatrists, in the near future, to interfere with ongoing brain activity with a reasonable temporal and spatial resolution to optimize clinical outcomes.

### A conceptual example of closed-loop tES-fMRI in a clinical population

**Fig. 5:**
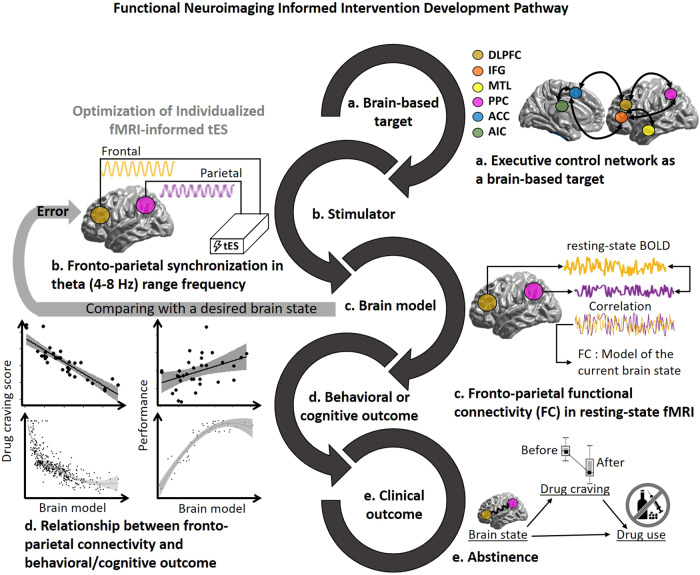
Exemplary functional neuroimaging-informed intervention development pathway. The pathway from the intervention (top) to clinical outcomes (bottom) is illustrated for the example of aiming for frontoparietal synchronization in drug addiction with a tES intervention in a closed-loop tES-fMRI system.

Here, we describe a closed-loop tES-fMRI study design to summarize the application of this system in neurological or psychiatric disorders. In Fig. 5, the pathway from selecting a stimulation target (top) to clinical outcomes (bottom) in a closed-loop tES-fMRI design is illustrated. This pathway includes the following main steps.

- Functional target: Abnormalities of executive control network (ECN) functions are reported in many neurological and psychiatric disorders (e.g., tinnitus [170], migraine [171], multiple sclerosis [172], schizophrenia [173, 174], depression [175, 176], anxiety [177, 178], obsessive-compulsive disorder (OCD) [179], and addiction [180]), and studies suggest that transcranial electrical stimulation (tES) methods are suited to modulate the ECN and its connections with other parts of the brain, especially with respect to frontoparietal connectivity [181–188]. Therefore, frontoparietal synchronization is a candidate target.
- Stimulator: Inspired by previous frontoparietal synchronization studies [185, 186], tACS in the theta (4–8 Hz) frequency range (as an initial value for the first iteration) to induce synchronized brain oscillations between frontal and parietal regions is considered as a promising intervention. For modulating frontoparietal synchronization, dual-site high-definition (HD) electrodes could be suited for targeting frontal and parietal nodes in the ECN. The first stimulation site can be determined based on studies that report an important role of the right DLPFC in executive functions. The center coordinate would be F4 in EEG 10 20 international system, and four surrounding electrodes (F1, F5, AF3, FC3) would be placed around F4, which results in a 4×1 montage with the center over the right DLPFC. The second stimulation site for placing another set of 4 × 1 electrodes would be defined based on the parietal brain region connected to the right DLPFC, obtained by fMRI using a task that activates the cognitive process of interest (e.g., the drug cue reactivity task in a group of participants with substance use disorders as suggested by [21]). Stimulation targets are shown by circles in Fig. 5b (circle colors represent frontal (brown) and parietal (purple) regions as the stimulation targets).
- Measure of the brain state: A biomarker is extracted from the predefined targets. Functional connectivity (FC) (i.e., correlation coefficient) between BOLD time series extracted from the frontal and parietal target regions can be considered as such a measure. This FC can be measured during the application of stimulation or immediately after the stimulation block (during resting-state or task-based fMRI recording). An optimization algorithm (e.g., Bayesian or multiplex optimization) will identify the optimal stimulation frequency and phase difference for stimulation targets (optimal parameters of stimulation may have different values for each site of stimulation as suggested by [189]) to enhance functional connectivity (by maximizing the connectivity or minimizing the differences between ongoing state and a desired value) between frontal and parietal regions in each iteration of optimization for each individual. It has been expected that the physiological state parameters are getting closer to the target value by adjusting stimulation parameters.
- Behavioral/cognitive outcomes: The extracted fMRI measure should explain a significant amount of variance of behavioral/cognitive changes induced by the intervention, and ideally, the actual brain state can be identified as reflecting the mediating variable that conveys the causal effect of the intervention on the behavioral outcome measure. In the case of application of frontoparietal tES in a group of participants with substance use disorder, drug craving, or performance in a cognitive task might serve as behavioral and cognitive outcomes, and FC between frontal and parietal regions is expected to significantly correlate with these behavioral/cognitive outcomes. However, this relationship/correlation might not always be linear; nonlinear relationships between measured brain state and behavioral or cognitive outcomes have been described.
- Clinical outcome: It is expected that the target engagement measure directly or indirectly (mediated by behavioral/cognitive outcomes in a complex chain of causation as suggested by [190]) contributes to clinical outcomes, such as abstinence. For example, the strength of the frontoparietal connection as a measure of brain state (as defined in part c) is correlated with drug craving and consumption.

## Conclusion

In this study, we reviewed emerging concepts of integrating NIBS with neuroimaging data (tES and fMRI in particular) in a real-time closed-loop approach. In sum, in a closed-loop brain stimulation system that can help to accelerate dose titration based on individualized actual brain state and a desired target value, there are two main models to be defined and parameterized; (1) the Brain Model that connects recording of the brain signals with a biomarker and clinical/behavioral outcomes, and (2) the Stimulation model that connects mechanisms of action of stimulation and its parameters (Fig. 1). The model parameters and the loop between these two models can be optimized following advancements in software innovations to enhance the quality of the analyses and the optimization and hardware innovations to make the sensors and stimulators more reliable, valid, and accurate. Despite the recognition of the potential benefits of the closed-loop approach, several challenges have to be overcome, which are discussed. The applicability of closed-loop tES-fMRI to patients with psychiatric or neurological disorders in clinical trials and eventually clinical practice critically depends on the (1) identification of validated target engagement and treatment response biomarkers for modeling actual brain states that correspond with behavioral and clinical outcomes and (2) extension of our understanding of the simulation model and its parameters based on the growing body of tES-fMRI studies. Experimental closed-loop tES studies have the potential to extend knowledge on both brain and stimulation models. A multi-site international collaboration between researchers, clinicians, and the industry can help to harmonize protocols for establishing closed-loop tES-fMRI equipment, data collection/sharing platforms, and optimization algorithms to collectively unsolved this complex puzzle. Although this approach was developed for tES, it has potential applicability for all interventions which allow rapid adaptation, including other brain stimulation protocols, such as combinations of TMS [191] or transcranial ultrasound stimulation (TUS) [17] with concurrent fMRI or other modalities like EEG or local pharmacotherapy interventions [192]. We hope that this paper provides a roadmap for this multidisciplinary collaborative partnership.
